# Perspectives on Human Hearing Loss, Cochlear Regeneration, and the Potential for Hearing Restoration Therapies

**DOI:** 10.3390/brainsci10100756

**Published:** 2020-10-20

**Authors:** Patricia M. White

**Affiliations:** Department of Neuroscience, Ernest J. Del Monte Institute for Neuroscience, University of Rochester Medical Center, 601 Elmwood Ave, Rochester, NY 14642, USA; Patricia_White@urmc.rochester.edu

**Keywords:** cochlear regeneration, hair cell, hearing restoration, hearing loss, noise damage, deaf perspective

## Abstract

Most adults who acquire hearing loss find it to be a disability that is poorly corrected by current prosthetics. This gap drives current research in cochlear mechanosensory hair cell regeneration and in hearing restoration. Birds and fish can spontaneously regenerate lost hair cells through a process that has become better defined in the last few years. Findings from these studies have informed new research on hair cell regeneration in the mammalian cochlea. Hair cell regeneration is one part of the greater problem of hearing restoration, as hearing loss can stem from a myriad of causes. This review discusses these issues and recent findings, and places them in the greater social context of need and community.

## 1. Introduction: The Prevalence, Cost and Impact of Hearing Loss

Worldwide, half a billion people have significant hearing loss, defined by the World Health Organization as auditory thresholds greater than 40 decibels (dB) in both ears [1]. This large number underestimates the broader prevalence of hearing impairment, which further includes individuals with unilateral hearing loss, difficulties discriminating speech in noise, and tinnitus. While 2–3 out of every thousand children exhibit hearing loss in their first year [2], the prevalence among adults over seventy years of age increases to 50% [3]. Among the active military, up to 60% of forward troops may experience hearing impairment [4]. Together, tinnitus and hearing loss affect ~17% of newly disabled soldiers [5]. Treatments for hearing impairment and disability payments cost the United States Veterans’ Administration over three billion dollars annually [5]. Even so, it is estimated that less than 20% of all individuals with hearing loss receive medical treatment [2]. Older adults who depend on oral language and who do not treat their acquired hearing loss are at increased risk for neurodegenerative dementia [6,7,8,9], further increasing its cost to individuals, families, and society.

The majority of individuals who acquire hearing loss as older adults lived for decades with oral language; for them its impact is disabling. Their hearing loss correlates with higher levels of morbidity, depression, and social isolation [10]. Hearing loss is defined by auditory thresholds, which are the softest sounds an individual can hear at a given frequency. Treatments include hearing aids for individuals with mild to moderate (up to 69 dB threshold increase) hearing loss, and cochlear implants for individuals with severe or profound (>70 dB) hearing loss [2]. These treatments can improve patients’ effective hearing thresholds, but can fall short in improving directional hearing and hearing in background noise. Many adjust to acquired hearing loss by lip-reading rather than seeking treatment, often because of social stigma, the cost of care, and other hurdles [11]. Lip-reading alone enables the identification of 12–20% of spoken words by an average untrained observer [12,13]. Relying on lip-reading likely interferes with medical care, education, and professional development. Free-form oral conversations within a large group are particularly challenging for lip-readers, due to the lack of visual cues to help them focus on who is speaking. Despite the difficulties of lip-reading, many patients find hearing aids difficult or unpleasant to use [14]. This drives the search for a biological treatment that would restore hearing to those who find its loss disabling.

Hearing loss may be classified as conductive or sensorineural. Sound waves enter the outer ear and are transmitted to the inner ear, the cochlea, through the auditory ossicles of the middle ear. Injuries, infections, or biological processes that impact middle ear function cause conductive hearing loss, whereas processes that impact cochlear function cause sensorineural hearing loss. This review will focus on causes of sensorineural hearing loss and recent progress in cochlear regeneration.

## 2. Cochlear Function

Information about sound is encoded by the cochlea and transmitted to the brain, and hearing loss occurs when this neural transmission is reduced or interrupted. Sound waves enter the cochlea through the oval window and travel through the perilymph fluid column, which acts as a frequency filter. Higher frequency sounds have a shorter wavelength and impact the basal cochlea, whereas lower frequency sounds have longer wavelengths and impact apical regions. Outer hair cells (OHCs) respond to and amplify vibrations to increase shear forces along the tectorial membrane, which stimulates the inner hair cells (IHCs). Hair cell (HC) activity is continuous and energetic. It is sustained by a unique ionic differential between the endolymph, which is high in potassium and low in sodium, and the perilymph, which is more similar to extracellular fluids. Called the endocochlear potential, this electrical gradient is maintained by a potassium recycling chain, where potassium excreted by HCs is taken up by neighboring supporting cells, and then transported to the cells of the stria vascularis. Strial cells lining the endolymph restore potassium to the endolymph. Loss of OHCs or reduced strial activity both impact the activity of IHCs and increase hearing thresholds.

IHCs encode sound information and transmit it to Type I spiral ganglion neurons (SGNs) through changes in the activity of specialized ribbon synapses. Type I SGNs comprise over 90% of total SGNs and receive input from IHCs; the more rare Type II SGNs innervate OHCs. Each IHC signals to up to 30 SGNs, depending on frequency and species, and each SGN receives auditory input from a single synapse. Type I SGNs are classified into three groups by gene expression, which encode auditory signals with low, medium, or high spontaneous discharge rates [15]. They project to the cochlear nucleus, which is the first relay for auditory information in the brainstem. OHC and SGN activity can be negatively regulated by efferent projections arising from the olivary complex, which protect the cochlea from traumatic noise [16]. Loss of auditory synapses or reduced synchrony in their firing are two mechanisms thought to underlie hearing impairments such as difficulties in hearing speech in background noise [17].

## 3. Causes of Sensorineural Hearing Loss

Hearing loss may arise from a myriad of causes, including aging, genetic variation, infection, and exposure to ototoxins or noise. It is intuitive that different kinds of insults could impact the chain of auditory transmission in different ways, resulting in the same impairment through different means. Cochlear cells express a number of proteins uniquely required for hearing, and genetic variants in those proteins can cause non-syndromic hearing loss. Other proteins may be necessary for hearing, and also function in other organs. Variants in those genes can cause syndromic disease, in which hearing loss is only one aspect. More than 120 genes for non-syndromic hearing loss have been identified, along with 55 genes for syndromic hearing loss [18]. For families with hearing loss variants, knowing the specific sequences underlying their hearing loss allows medical providers to predict if their hearing loss is part of a syndrome that may lead to further comorbidities, including heart [19], thyroid [20], or visual dysfunction [21]. Genetic variants may also potentiate hearing loss from noise damage [22], drug damage [23], or aging [24]. It is important to note that even within families, the same genetic variants can lead to hearing loss on different time scales and to differing extents, likely due to the presence of genetic or environmental modifiers.

Infectious pathogens can drive hearing loss. As a sequela from disease, this may occur to either children or adults. Perinatal or prenatal infection by so-called TORCH pathogens, which include rubella, cytomegalovirus, and other viruses, is associated with childhood hearing loss [25]. Adults who contract diseases as varied as mumps [26], AIDS [27], or meningitis [28] may also develop hearing loss. While it is likely that diseases impact the cochlea in different ways, inflammation of the lateral wall and stria vascularis are thought to be a modifiable mechanism of hearing loss from infection. In animal models, reductions in the endocochlear potential leads to concomitant threshold increases [29]. In the clinic, sudden sensorineural hearing loss is treated with anti-inflammatory glucocorticoids [30]. For some patients, this treatment correlates with hearing restoration [31,32]; however, there are also reports of individuals who recover spontaneously [33].

Platinum-based chemotherapies and certain antibiotics are ototoxic and cause hearing loss (reviewed in [34]). Ototoxic medications may be used if necessary to save the life of the patient. At the doses required for clinical efficacy, they can kill HCs and the surrounding supporting cells [35]. In addition to prescribed medications, drugs of abuse containing acetaminophen can be ototoxic when taken in excess [36].

Noise exposure is a significant cause of hearing loss, estimated to affect 10 million adults in the United States [37]. Traumatic noise exposure can come from loud percussive insults, such as explosions and gunfire, low- or mid-frequency noise, such as industrial or construction noise, or recreational pursuits, including loud music. Sustained exposure to noise greater than 85 dB in occupational settings highly correlates with hearing loss among workers [37]. Hearing loss from noise exposure is characterized by higher thresholds in a subset of high-frequency sounds (3–6 kHz), sometimes referred to as a “audiometric notches” [38]. These are outside the frequency range of the noise exposure. Basal OHCs, which respond to higher frequencies, are sensitive to insult and are often lost in animal studies of noise damage [39,40] as well as studies of human cadavers [41]. In addition to cellular losses, noise can destroy SGN synapses [42,43] through glutamate excitotoxicity [44]. Certain subclasses of SGNs are thought to be more sensitive to noise damage [45], which could affect the perception of speech in noise [46]. Finally, efferent regulation of the cochlea is also modified by noise exposure [47], although the consequences of these changes for hearing function are poorly understood.

## 4. Spontaneous Cochlear Regeneration

Over thirty years ago, two groups serendipitously discovered that birds spontaneously regenerate damaged auditory HCs [48,49]. A time course analysis of HC death after gentamycin administration in hatchling chicks revealed that two weeks after HCs were lost, a nearly normal complement had been regenerated [50]. Concomitantly, experiments using traumatic noise to identify where sound frequencies map on the bird cochlea, called the basilar papilla, also discovered nascent HCs ten days after injury [51]. Further experimentation determined that adjacent supporting cells divided rapidly after HC loss, as they could be labeled by thymidine analogues [52]. Days later, labeled HCs were observed, suggesting that they differentiated from the dividing supporting cells. The restoration of hearing thresholds was complete five months later, correlating with HC maturation [53]. Notably, this study did not measure hearing recovery between 4 and 20 weeks. In a later experimental series, songbirds recovered their ability to discriminate between vocalizations seven weeks after damage [54].

**Figure 1:**
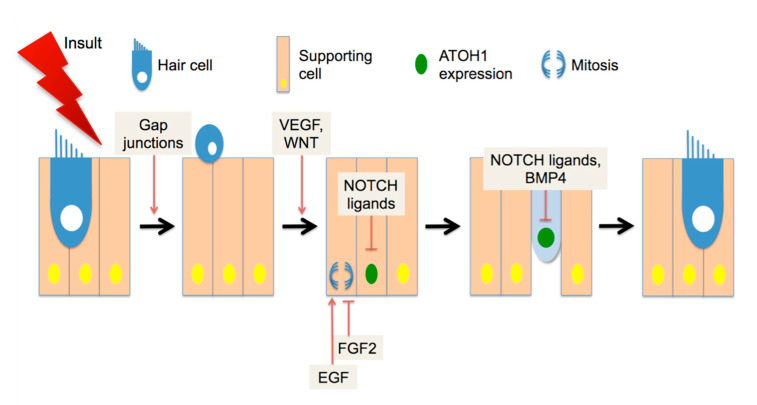
Factors in avian auditory regeneration. Hair cells (HC, blue) are surrounded by supporting cells (tan) in the basilar papilla. After noise or ototoxin exposure, HCs are expelled from the epithelium. VEGF (vascular endothelial growth factor) and/or WNT family factors have been implicated in both supporting cell proliferation and HC differentiation, with the latter process requiring ATOH1 (atonal homologue 1) expression (green nucleus). NOTCH ligands act to down-regulate ATOH1 expression. EGF (epidermal growth factor) ligands and FGF2 (fibroblast growth factor) have opposing effects on proliferation (indicated in blue). After the nascent HC is specified, BMP (bone morphogenic protein) ligands are thought to inhibit further differentiation through the induction of ID (inhibitor of differentiation) proteins. See the text for references.

What signaling pathways regulate avian regeneration? Gap junction communication (Figure 1) is required for supporting cells to expel HC corpses from the epithelium and fill in the gaps left by their loss [55]. Pharmacological studies on developing and regenerating basilar papilla implicated Wnt/β-catenin activity as positively regulating both organ size and number of HCs [56], suggesting that this factor may act early in the regenerating pathway (Figure 1). The Wnt family of ligands signals through the Frizzled receptors to stabilize intracellular β-catenin activity. Evidence for Wnt/β-catenin activity in avian regeneration has also been found in transcriptome analysis of regenerating basilar papilla [57]. Similarly, vascular endothelial growth factor (VEGF) has been shown to promote supporting cell proliferation and HC differentiation in cultures of basilar papilla (Figure 1), and pharmacological inhibitors to that pathway also block both responses [58]. Interestingly, the same study showed that VEGF is expressed by HCs but not secreted. During apoptosis, HCs appear to shed VEGF into the tectorial membrane, where it can interact with receptors on supporting cells [58]. This puts VEGF at the time and place to be the initial regeneration signal, which may stimulate both supporting cell proliferation and HC differentiation.

Other investigations have shown that HC differentiation and supporting cell proliferation are further regulated independently. To initiate differentiation into HCs, supporting cells or precursor cells must express the basic helix-loop-helix (bHLH) transcription factor ATOH1 [59]. In experiments with rodent cochleae, ATOH1 is both necessary [60] and sufficient [61] for the induction of HC differentiation in some cochlear cells (but see [62] for limitations). ATOH1 protein positively regulates its own transcription by interacting with enhancer elements in its promoter [63]. Transcription of *Atoh1* is negatively regulated by NOTCH signaling [64]. ATOH1 activity is also negatively regulated at the protein level, through binding partners lacking a DNA binding domain such as the ID [65] and HES proteins, the latter of which are also NOTCH effectors [66]. The expression of the NOTCH ligand DELTA1 is one of the earliest differentiation events after ATOH1 induction in regenerating avian HCs [67]. During chick basilar papilla development, DELTA1 and JAG2 expression in nascent HCs drive NOTCH activity in surrounding cells, promoting a supporting cell fate [68]. Inhibition of NOTCH during regeneration promotes ectopic HC differentiation [69], indicating that NOTCH negatively regulates the specification of HCs (Figure 1). After specification through ATOH1 expression has occurred, further differentiation may be inhibited by the presence of BMP4 (Figure 1), which promotes ID expression [70]. HC specification initiates rapidly during regeneration and continues for several days [71]. In contrast, supporting cell proliferation initiates later, and may generate either two supporting cells or one supporting cell and one HC [72]. Proliferation requires signaling through the epidermal growth factor (EGF) receptor family [73], and is negatively regulated by fibroblast growth factor (FGF) ligands [74]. In addition to these pathways, transcriptomic experiments have identified modulation of additional pathway effectors during regeneration [57,75], suggesting a complicated interplay of positive and negative signaling.

Spontaneous HC regeneration is also observed in cold-blooded animals, including fish [76]. In addition to their inner ear, fish harbor HCs in organs of the lateral line on the surface of their bodies, which detect changes in water movements. Located in cell clusters called neuromasts, these HCs can be easily monitored through time-lapse microscopy. Neuromasts are dome-shaped, with a central group of HCs atop a base of supporting cells, surrounded by non-sensory mantle cells [77]. Rapid HC loss can be induced by bathing the fish in neomycin or other ototoxic chemicals [78]. Within three days, the adjacent supporting cells will generate new functional HCs [78]. Differentiation always follows supporting cell mitosis, and the paired daughter cells symmetrically adopt either a HC or supporting cell fate. The latter division can be followed by a HC differentiation division [79]. These data are interpreted to mean that neuromast-supporting cells contain a mix of HC progenitors and self-renewing stem cells. The HC progenitors are located in the center of the neuromast, whereas the stem cells are located at the anterior and posterior poles [79]. Strikingly, the surrounding mantle cells act as long-term quiescent stem cells for the neuromast, in addition to providing specific niche signals to maintain the self-renewing stem cells [80]. Analysis of the regulation of neuromast regeneration reveals spatially segregated, parallel signaling pathways to control proliferation, differentiation, and organ size. In the undamaged neuromast, newly differentiated HCs express ATOH1 and NOTCH ligands, which repress HC differentiation by progenitor cells. FGF3 is expressed by central supporting cells. Both FGF3 and NOTCH independently suppress WNT signaling, which is required for proliferation of progenitors and stem cells [79,81]. Immediately after HC loss, the JAK1/STAT3 pathway becomes activated [82,83], and NOTCH and FGF3 signaling are inactivated. These events permit the induction of WNT signaling and subsequent regeneration. It should be emphasized that NOTCH has multiple regulatory roles in neuromast regeneration, inhibiting both differentiation and proliferation.

## 5. Recent Developments in Mammalian HC Regeneration

In pursuit of therapeutics to restore hearing from drug or noise damage, investigators have focused on the cellular events that could regulate the generation of new HCs from supporting cells in the mammalian cochlea. Here the mouse has become a commonly used model system for human hearing loss. Mice are less expensive to maintain than larger rodents such as guinea pigs. They can be maintained as congenic lines with well-described damage characteristics. Finally, they may be manipulated genetically to assess gain and loss of function of candidate pathways, permanently mark cell types, or perform cell-specific ablation studies. Like other rodents, mice are born before the onset of hearing, which occurs around two weeks of age (post-natal day 14, or P14). Interestingly, the immature neonatal mouse cochlea retains a limited ability for regeneration, both in vitro [84] and in vivo [85]. Moreover, the mammalian vestibular system retains a limited capacity for regeneration (see [86] for review). Understanding auditory regenerative capacity and how it changes during maturation are under intense investigation. The similarities and differences between cochlear development and regeneration were recently reviewed [87]. Here I will focus on new findings that address progenitor populations and signaling pathways.

**Figure 2:**
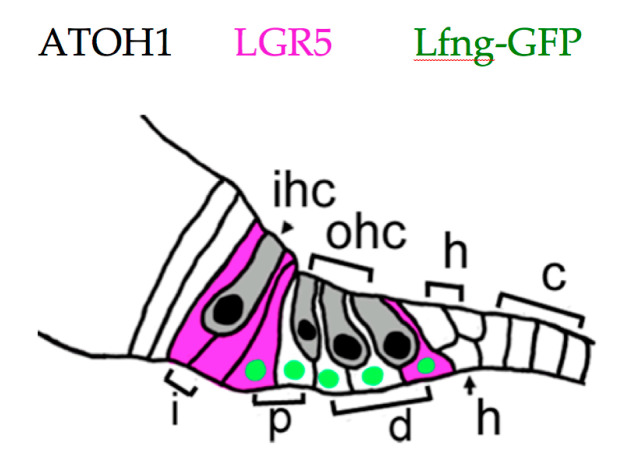
Neonatal cochlear supporting cells. The inner hair cell (ihc) and outer hair cells (ohc) are depicted in gray, with ATOH1+ nuclei in black. Interphalangeal cells (i), pillar cells (p), Deiter cells (d), Hensens cells (h), and Claudius cells (c) are all indicated. LGR5+ (Leucine-rich repeat containing G-protein coupled receptor 5) cells are indicated in magenta, and Lfng-GFP+ cells with green nuclei. All supporting cells express SOX2 (Sry-box 2, not shown). See text for references.

Cochlear progenitors may be isolated, purified, propagated in sphere culture, and differentiated into HCs, supporting cells, neurons, and glia. Using these methods, multiple labs have found that supporting cell populations which include interphalangeal cells, the inner pillar cell, and the third Deiter cell (Figure 2) have robust capacity for proliferation and HC generation [84,88,89]. These cells express the WNT response gene *Lgr5* at birth [88,89], and divide when stimulated by either WNT ligands or agonists [88,89] or by SHH (Sonic hedgehog) agonists [90]. The ability to be propagated as spheres or to generate HCs declines with age, and is largely lost by P30 [91,92]. This matches results from tissues obtained from adult human cochlea, where only a single sphere was cultured from ten individual samples [93].

Progenitors for cochlear regeneration can also be studied in organ culture or in vivo, by stimulating regeneration through HC ablation, activating candidate pathways, or combining multiple approaches. Cell-specific ablation may be achieved through genetic expression of the human diphtheria toxin receptor (DTR), followed by injection of diphtheria toxin [85]. HC loss is observed 2–4 days later, and supporting cell proliferation and conversion to HCs occurs largely in the cochlear apex [85]. Lineage tracing has revealed that pillar and Deiter cells comprise the most precursors to HCs, and conversion is not exclusive to LGR5+ cells [94], suggesting that HC differentiation capacity may not be restricted to sphere-forming cells.

Many early efforts on understanding supporting cell to HC transdifferentiation focused on the effects of manipulating NOTCH signaling on supporting cells in the cochlea (see [95] for a comprehensive review). Inhibition of γ-secretase, an enzyme necessary for NOTCH signaling, or addition of anti-NOTCH1 blocking antibodies both interfere with NOTCH1 signaling, and promote the expression of ATOH1 and early HC markers in apical neonatal cochlear supporting cells in culture [64,96,97]. NOTCH signaling promotes the expression of the transcription factor SOX2 [98], which marks cochlear sensory progenitors from embryonic day 12 (E12.5, [99]) and supporting cells through adulthood. SOX2 is required for *Atoh1* expression; however, it concomitantly promotes the expression of the ATOH1 antagonists such as *Hes* and *Id* family members [100,101]. This counter-intuitive process is called an incoherent feed-forward loop [102]. It can drive a pulse-like accumulation of the target, ATOH1, when SOX2 expression is abruptly down-regulated through the interruption of NOTCH signaling. Indeed, use of the haplo-insufficient Sox2-CRE^ERT2^ mouse line to label supporting cells appears to promote both proliferation and hair cell conversion through WNT and NOTCH-dependent processes [103]. Maintaining NOTCH signaling in vivo in neonatal apical supporting cells blocks spontaneous HC regeneration in the DTR model [104]. However, expression of ATOH1 alone in supporting cells without HC loss enables only partial induction of the HC fate [62], indicating that other factors are needed for differentiation. By postnatal day 6 (P6), some components of the NOTCH signaling pathway become down-regulated [105], and supporting cells no longer induce ATOH1 in response to NOTCH signal blockade [97]. Not surprisingly, the transcriptional profile of adult supporting cells is highly distinct from the neonatal stage [106]. Taken together, these data support the conclusion that NOTCH manipulation has the potential to regulate the supporting cell to HC conversion in mammals before the onset of hearing, but is likely insufficient to drive such activity in adults.

WNT signaling was also implicated early in the specification of the otic placode [107,108] and in promoting *Atoh1* transcription [109]. Analysis of WNT signaling often focuses on the canonical pathway, where WNT signals stabilize the effector β-catenin. Signaling can thus be mimicked by over-expressing a constitutively active β-catenin (CATNB) in responding cells, or through the application of WNT agonists like CHIR 99021 [110]. Co-expression of CATNB and ATOH1 in neonatal supporting cells promotes more differentiation of HC-like cells compared to ATOH1 alone [111], suggesting that CATNB also activates co-factors of ATOH1. These co-factors may include the up-regulation of GATA3 and POU4F3, and the down-regulation of CDKN1B [112]. As with NOTCH signaling, blocking WNT also inhibits spontaneous HC regeneration induced in the DTR model [113]. WNT activation through agonist application stimulates robust proliferation in neonatal SOX2+ supporting cells in vitro, and promotes HC differentiation from NOTCH blockade [114]. As the cochlea matures, however, both proliferation and the ability to support HC differentiation from supporting cells in response to exogenous WNT becomes lost [114]. Thus, while in the neuromast interruptions in both NOTCH and FGF3 signaling promote WNT expression, in the immature mouse cochlea, WNT activation and NOTCH inhibition appear to act in parallel, with both signals required for HC differentiation.

Investigations into mammalian cochlear regeneration also assess the effects of signaling pathways not implicated by studies of chick and fish. For example, SHH specifies ventral otic structures, including the cochlea, during the otic vesicle stage of development [115]. At later stages, SHH signaling inhibits prosensory formation [116]. During the neonatal stage, proliferation and HC differentiation during spontaneous regeneration in vitro from neomycin treatment are increased by the expression of an activated SHH receptor, Smo-OE [90]. Understanding the effects of SHH is complicated by the presence of opposing gradients of GLI proteins in the cochlea [117]. GLI proteins are concentration-dependent SHH negative modulators which shape the morphogenic response to SHH.

ERBB2 signaling is an additional candidate pathway under investigation in mammals. ERBB2 is one of four members of the EGF receptor family, all of which are expressed by mammalian supporting cells throughout life [118]. The family of EGF receptors was first implicated in auditory regeneration in the chick, where incubation with inhibitors to the family was shown to block supporting cell proliferation [73]. Moreover, exogenous WNT signaling has been shown to up-regulate ERBB2 as well as other pathway components in cochlear supporting cells [114]. Expression of a constitutively active form of ERBB2 (CA-ERBB2) was shown to drive SOX2 down-regulation in vitro, and promote ectopic HC formation in vivo through a mechanism that was non-cell autonomous [119]. Notably, the CA-ERBB2 allele specifically activates PI3K [119,120], which is also a downstream effector of the VEGF receptor. Thus, these reported effects may also be due to a convergence of signaling pathways.

## 6. Hearing Restoration after Noise Damage in Adult Mammals

While progress has been made towards a better understanding of HC differentiation in the immature mouse cochlea, the problem of hearing restoration remains. Several groups have assessed the effects of driving ATOH1 expression or blocking NOTCH activity in the cochlea after noise damage, using different approaches. Gene therapy, consisting of intra-labyrinth injection of adenoviruses driving expression of the HC determinant Atoh1, has been proposed as means of restoring hearing in deafened adults [121]. A clinical trial by Novartis using such a preparation, named CGF166, has recently concluded, with results still in preparation [122]. In a second approach, inhibitors to γ-secretase were applied to the round window of animals after traumatic noise exposure [123,124]. This approach seeks to promote regeneration by interfering with NOTCH signaling. One experimental series revealed a partial recovery of low frequency thresholds (~8 dB out of ~40 dB threshold shift) in mice fourteen days after noise exposure, with a concomitant improvement in HC numbers [123]. This study used the Sox2-CRE^ERT2^ mouse line, which may be predisposed to HC differentiation [103]. The other used guinea pigs as an experimental model, but only tested three animals [124]. Lastly, siRNA against the NOTCH effector *Hes1* have been introduced into the cochleae of guinea pigs after noise exposure [125]. This intervention drove improvements of 8–12 dB from an initial threshold shift of 60–80 dB, when compared to scrambled RNA controls. In this experiment, three frequencies showed significant improvements. The improvements were evident at 3 weeks post-noise and were maintained for 9 weeks. They also correlated with improved numbers of HCs. While highly promising, it is possible that all of these results represent an augmented repair of HCs rather than regeneration. Atoh1 viruses, γ-secretase inhibitors, and Hes1 siRNAs were introduced to the cochlea within three days after noise damage, which is prior to the second wave of HC apoptosis after noise damage [126]. Moreover, the functional improvements were rapid when compared to hearing restoration in regenerating birds [54].

Additional hurdles remain to the development of HC differentiation from supporting cells in adults. Adult supporting cells have significant transcriptional differences from neonatal supporting cells [91,106], suggesting the need for additional strategies besides ATOH1 expression. Indeed, multifactorial approaches are increasingly employed [112,127]. Alternatively, changes to the epigenetic landscape of supporting cells may improve ATOH1 responsiveness [128]. Epigenetics refers to chemical modifications on DNA or DNA binding proteins, which are stable over time and affect the availability of chromosomal regions for transcription (for review, see [129]). In addition to HC differentiation, replacing converting supporting cells remains an important consideration. Without sufficient numbers of supporting cells, long-term functionality is very likely compromised [130]. Sphere-forming capability from the adult human cochleae is quite poor when compared to adult human utricles, indicating a more stringent regulation of proliferation [93]. Recently, supporting cell and IHC proliferation were both demonstrated after the transduction of the oncogene MYC in conjunction with increased NOTCH signaling in adult cochlear cells [131]. The process required transient activation, as sustained activation drove significant levels of cell death [131]. Taken together, it is anticipated that multiple genes will need to be expressed or activated to drive HC regeneration, that their activity must be regulated in time, and that the group will likely contain a known oncogene. This may be effected through gene therapy, where viruses engineered to express specific genes are injected into cochlea [132], through the application of compounds like CHIR 99021, or some combination. Tailoring the approach to only affect organ of Corti cells will be crucial to avoid compromising cranial nerve function, for example by inducing schwannomae. While the problem is complex and many hurdles remain, it is exciting to contemplate regeneration strategies. Indeed, dozens of new companies are working to implement a variety of therapeutic approaches [133,134].

**Figure 3:**
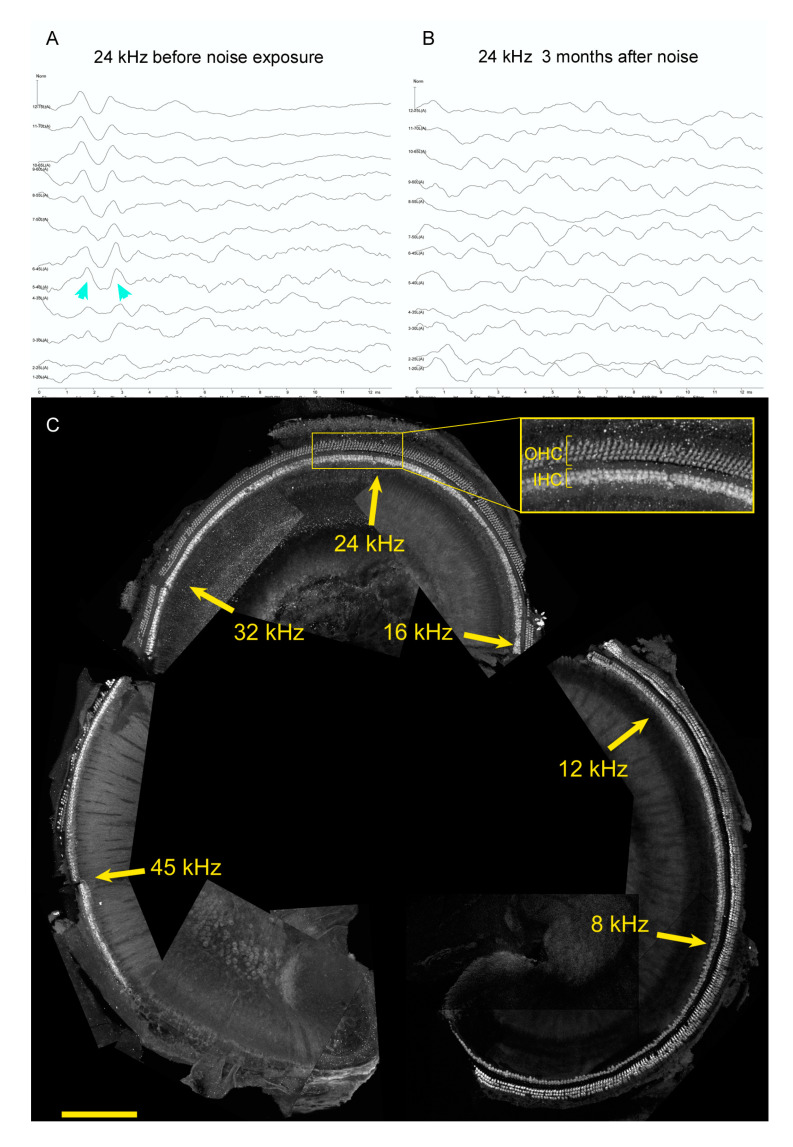
Hearing loss from noise does not require HC loss. (**A**,**B**) ABR to 24 kHz stimuli presented at 5 dB intervals, starting at 75 dB (top trace) and ending at 20 dB. Results from a test administered to a naïve mouse at 1 month of age (**A**) are compared to the same mouse three months after traumatic noise exposure (**B**). Cyan arrowheads indicate the threshold trace in (**A**); no waveforms are seen after traumatic noise. (**C**) Cochlea surface preparation from the same mouse euthanized after the testing shown in (**B**). Anti-MYO7 and anti-OCM immunostaining are both shown in white to reveal IHCs and OHCs. Approximate frequencies are indicated in yellow. The inset shows a high-power image of the region of interest, with OHC and IHC populations indicated. Size bar: 200 microns. Methods are from [135].

Lastly, restoring hearing after noise damage may require additional approaches beyond assessing pathways that promote HC differentiation. Figure 3 shows representative data for a four-month old genetic control mouse from a noise damage experiment (see [135] for methods and statistical analysis). The mouse was an F1 hybrid of CBA/CaJ mated to C57BL6/J, a cross that exhibits good hearing up to a year of age and youthful sensitivity to noise [136]. It was exposed to an octave band of noise, 8–16 kHz, at 110 dB for 2 h when it was one month old. Inspection of auditory brainstem responses (ABR) to 24 kHz stimuli prior to noise exposure (Figure 3A) and three months after noise exposure (Figure 3B) confirms that this traumatic noise exposure conferred large permanent threshold shifts. Similar results were obtained for 8, 12, 16, and 32 kHz (not shown). After euthanasia, one cochlea was microdissected, mapped, immunostained with antibodies to reveal HCs, and imaged on a confocal microscope. The images were assembled for cochleogram analysis (Figure 3C). Approximate frequency locations are noted on the montage. HC loss is evident in the ultrasonic regions around 45 kHz, but not in the mid or lower frequencies (Figure 3C, note inset). These data demonstrate that while HC loss is sufficient to drive hearing loss [137], it is only one mechanism that permanently increases thresholds after noise damage. Other mechanisms could include HC intrinsic damage, strial and supporting cell damage, changes in efferent function, and changes in spiral ganglion neuron responses. Experimental approaches that focus on the recovery of auditory function may thus yield complementary information to those that focus on HC regeneration.

## 7. Conclusions

Since the discovery of spontaneous HC regeneration and hearing restoration in birds after noise or drug damage, research efforts have improved our understanding of the cellular events that underlie this response. In particular, the progenitor cells and signaling pathways that enable the development of new HCs are becoming better understood. Efforts to extend these findings from bird and fish to the mammalian cochlea highlight the similarities and differences of these systems. Prior to the onset of hearing, the mammalian cochlear cells are plastic in their specification, but this capacity changes with maturity. There remain significant hurdles to overcome in pursuit of therapeutic solutions, especially for noise damage where it is clear that HC loss alone does not explain the loss of function. Furthermore, investigations will be needed to address hearing loss from other causes, especially infections.

It should be noted that any discussion on hearing restoration must occur with the understanding that this is not something desired by all individuals with hearing loss. In fact, there are communities of adults with hearing loss who view hearing restoration as cultural genocide [138,139]. This community particularly consists of, but is not exclusive to, those who were born with hearing loss and are not able to comprehend oral language with or without hearing aids or cochlear implants. This community of Deaf individuals who use sign language as a first language do not experience hearing loss as a disability. Like oral languages, signed language utilizes the brain’s core language network, including Broca’s and Wernicke’s areas [140,141]. Many deaf adults want to have deaf children like themselves and view themselves and their language as an ethnic community [142,143]. Medical therapies that seek to restore hearing have the potential to help many people who desire it, but must be placed in a broader public health context recognizing that a single solution is insufficient to address the many needs of this diverse community.
